# Our Journal

**Published:** 1883-11

**Authors:** 


### OUR JOURNAL.

The subscription list of the INDEPENDENT PRACTITIONER, already quite extended, is rapidly increasing. The journal goes to every section of our country, from Maine to the Gulf of Mexico, from the Atlantic coast to the Pacific slope. It has subscribers also in all parts of Europe, South America, Canada and Cuba. Advertisers will please note this fact.

Subscribers who are in arrears are respectfully requested to forward the amount of their indebtedness to the treasurer, Dr. C. E. Francis, 33 West Forty-Seventh Street, New York.

